# Long-term population monitoring of a territorial forest raptor species

**DOI:** 10.1038/s41597-020-0503-x

**Published:** 2020-06-01

**Authors:** María V. Jiménez-Franco, José E. Martínez, Iluminada Pagán, José F. Calvo

**Affiliations:** 1 Departamento de Ecología e Hidrología, Universidad de Murcia, Murcia, Spain; 2 Departamento de Biología Aplicada, Universidad Miguel Hernández de Elche, Elche, Spain; 3 Bonelli’s Eagle Study and Conservation Group, Murcia, Spain

**Keywords:** Animal behaviour, Conservation biology, Population dynamics

## Abstract

We provide field monitoring data of a territorial raptor (the booted eagle, *Hieraaetus pennatus*), that was intensively monitored over a period of 18 years (1998–2015) in a Mediterranean forested area of south-eastern Spain designated as a Special Protection Area (Natura 2000 Network) for this species. The data set compiles all the relevant information about the occupation of territories and nests, reproductive ecology, long-term monitoring of marked individuals and influence of parent’s colour morph on brood size. Several questions concerning the population ecology of forest-dwelling raptors and factors conditioning territorial occupancy, such as location cues or site fidelity, are addressed. This type of long-term population monitoring has high potential for replication, reuse and comparison purposes, providing insights for monitoring other long-lived, territorial species.

## Abstract (summary)

**Table:** 

Measurement(s)	Occupancy • reproduction • marked individuals • phenotypic annotation • color
Technology Type(s)	Sampling • visual observation method
Factor Type(s)	species • sex • territory • nest
Sample Characteristic - Organism	Hieraaetus pennatus • Buteo buteo • Accipiter gentilis
Sample Characteristic - Environment	mediterranean forest biome
Sample Characteristic - Location	Murcia

Machine-accessible metadata file describing the reported data: 10.6084/m9.figshare.12097776

## Background & Summary

Long-term population monitoring provides valuable insights into ecology, environmental change and the management of natural resources^1^. Since different factors affect a given population structure, population monitoring should establish systematic programmes to collect biological information in a consistent way, especially in relation to time and environment^2^. Monitoring should not be viewed as a stand-alone activity, but as a component of a larger process of conservation-oriented science or management^3^. We believe these standardized protocols on long‐term data management will be useful to wildlife managers, ecologists, and others to develop their management programmes and improve their ability to archive and share important ecological data^4^. Standardized monitoring programmes in species ranges developed by wildlife managers, would strengthen the management of both highly endangered as well as healthy populations^5^. For this reason, it is important that monitoring projects provide detailed guidance with broad recommendations for data collection, data management and examples which are useful for practitioners.

There are different examples of long-term monitoring programmes of wildlife, ranging from global^6^ to continental^7,8^ scales, and others that describe the population trends of single species^9^. Nevertheless, in most cases, there is a lack of standardized protocols between programmes^5^. Monitoring efforts of limited duration can result in partial or even biased information^10^, and the delayed detection of threatened viability and population changes. Therefore, the development of practical, affordable and broadly applicable methods for monitoring vertebrates with slow life-history traits remains a challenge for applied ecologists worldwide.

Monitoring on a long-term scale allows population trends, colonisations and extinctions to be ascertained. Changes in abundance (population trends) and occupancy (local extinctions and colonisations) are both important components of biodiversity change, and contribute in correlated but different ways to biotic change^11^. Moreover, breeding habitat selection is an important process since it must guarantee food and protection for a long breeding period, which is a critical time of the life cycle^12^, and may influence reproductive output. Notwithstanding the importance of population monitoring, it is not sufficient to simply document trends in time and space (e.g., population-specific rates of occupancy and reproductive success) without placing these trends in the context of long-term variability in a global change context^13^. Therefore, the study of recent population trends monitored worldwide provides useful knowledge to analyse global change processes. In this study, we provide census data on a non-invasive long-term (18 years, 1998–2015) monitoring programme of a booted eagle (*Hieraaetus pennatus*) population in a Mediterranean forested area located in the Natura 2000 Special Protection Area “Sierras de Burete, Lavia y Cambrón”, south-eastern Spain. The dataset^14^ includes information about nest and territorial occupancy, productivity, individuals’ identification as well as the colour polymorphism of booted eagle. Raptors properly act as “sentinels” of different local and large-scale environmental changes and global threats to biodiversity^15,16^, as in the case of the booted eagle in the study area^17–19^. Raptors are also sensitive to changes in land use, and are highly susceptible to local extinctions^20^. Therefore, long-term monitoring of raptor populations makes it possible to identify conservation threats to birds and their habitats, making them an ideal tool for establishing conservation measures^21^.

**Fig. 1:**
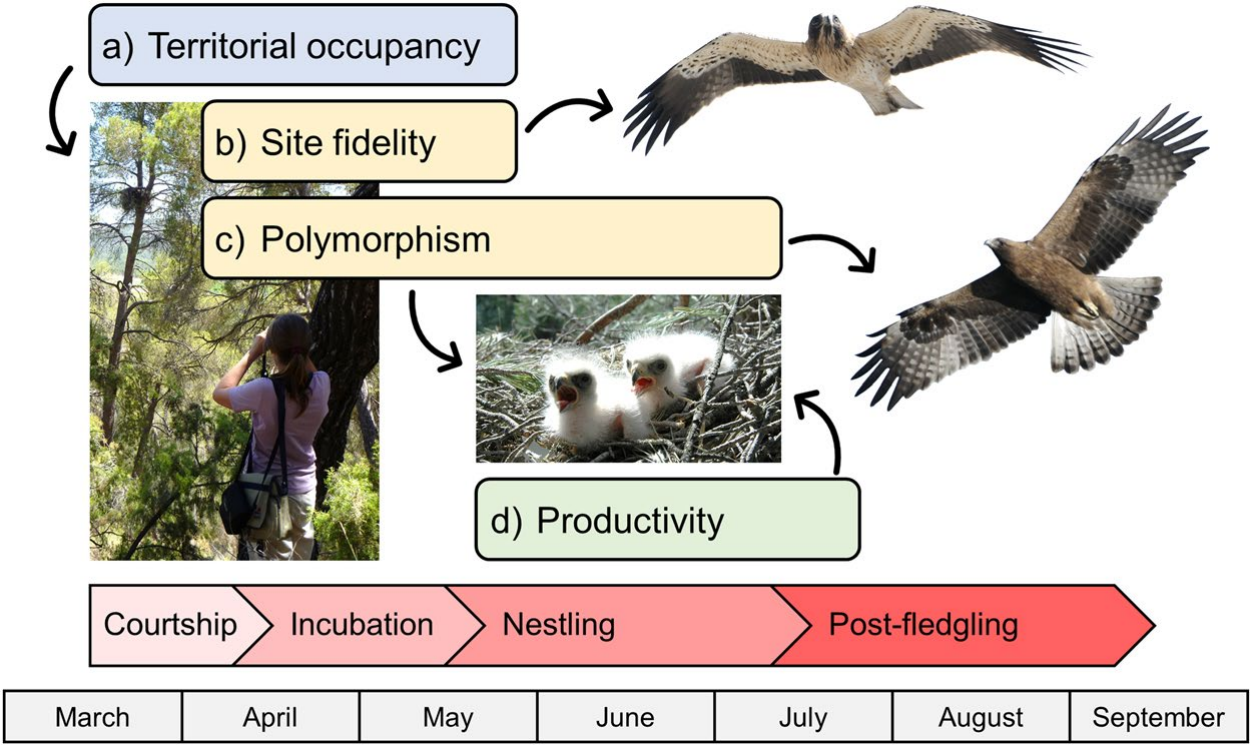
Biological processes recorded in the monitoring programme of booted eagle population in relation to timeline during the breeding period. This framework generates the main four types of data described in this study (**a–d**, Tables Occupancy, Marked-individuals, Polymorphism and Productivity, respectively^14^), where one or more nests are the simplest sampling units that constitute territories: (**a**) territorial occupancy recorded by visual observation of nests; (**b**) site fidelity recorded by individual identification of breeding individuals using drawings and photographs; (**c**) colour polymorphism of breeding individuals individually identified and fledglings; (**d**) productivity represents the number of fledglings per monitored pair recorded by optics or climbing nests. Photo credits: María V. Jiménez-Franco (nest); Carlos González Revelles (eagles).

Although the recent literature highlights the significant benefits of wildlife research programmes using systematic data management^3,13^, the practical benefits of such management are still underused by wildlife researchers^4^. Our objective in compiling the data described herein, using forest raptor ecology as a study system, was to provide practical guidelines for studying population ecology during the reproductive period for raptor field research (Fig. 1). We provide justification, definitions, instructions and examples of systematic forest raptor data collection in a Mediterranean forest, as well as ecological processes and references of ecological studies that can be obtained from the collected information along years. We consider this study may act as a reference to be replicated by wildlife managers and researchers in territorial raptor monitoring since most research protocols of birds of prey (raptors and owls) have similar data needs: the identification of distinct nesting sites (nesting-platforms, cliff-nesting, cavities, any elevated natural or man-made structure, etc.) and territories^12,22,23^, the collection of reproductive data at territories, and identification of individuals (breeding pairs and nestlings) within and across years^13^. Moreover, we illustrate ecological and population hypotheses through scientific studies that are flexible enough to be implemented with other animal taxa and at other spatio-temporal scales. The implementation of these standardized monitoring programmes will allow international collaboration and a comparison of estimated demographic parameters, which are necessary for managing long-lived species such as raptors^5^.

## Methods

### Study area and study species

The forest ecosystem studied is situated in the centre of the province of Murcia, south-eastern Spain (38°00′N, 1°45′W), with an area of 10,000 ha and declared as a Special Protection Area for wild birds (SPA Sierras de Burete, Lavia y Cambrón; “ES0000267”). The climate is dry Mediterranean with an annual precipitation of c. 400 mm and mean temperature of 17 °C. The mountainous landscape (between 550 and 1234 m above sea level) contains large forest patches dominated by Aleppo pine *Pinus halepensis* on hillsides, small groves of *Quercus rotundifolia* on the highest peaks, and extensive agricultural areas in the valleys with mostly dry-land crops including vine, olives, almonds and cereals^24^. The area is designated as a SPA under Annex I of the EU Directive 2009/147/EC relating to the conservation of wild birds, including the booted eagle (*Hieraaetus pennatus*), the short-toed snake eagle (*Circaetus gallicus*), the eagle-owl (*Bubo bubo*) and the red-billed chough (*Pyrrhocorax pyrrhocorax*). Moreover, a part of the studied area, “Sierra de Lavia”, has been included in the list of sites likely to be included in the list of Special Areas of Conservation (SAC; “LIC ES6200021”). The territory proposed for inclusion as SAC occupies 10% of the total SPA. Although the main activities in the study area are quarrying and hunting, the forested areas were not greatly disturbed by commercial exploitation (only forest management and regeneration projects, both on public and private land), so most of the forest can be considered mature, making it a suitable area to study nest dynamics and raptor ecology over a period of time^25,26^. The results obtained for the studied area can be extrapolated to other Mediterranean areas and European forest systems harbouring birds of prey^27,28^. The forest raptor population studied, the booted eagle (mean body mass ca. 691–973 g in males and females^29^), is a trans-Saharan migrant raptor, which arrives in Europe in late March and leaves in September. In our study area, this species exploits a wide range of prey^30,31^. The conservation status of booted eagle is vulnerable^32^, power lines and killing being the main known threats^33^. This species shows a strong territorial behaviour^34,35^ and breeding phenology, and may also alternate territories in different years^36^ with other forest raptor species, such as common buzzard (*Buteo buteo*; mean body mass ca. 662–800 g in males and females^37^) and the northern goshawk (*Accipiter gentilis*; mean body mass ca. 912–1,137 g in males and females^38^). These species are sedentary in the study area with local winter movements^39^.

### Field work

The data for this study of the ecology of a forest raptor species were obtained by non-manipulative observational sampling of the environment. The booted eagle population was monitored during the reproductive period (March-August) (Fig. 1) over a period of 18 years (1998–2015). The data compiled included territorial and nest occupation, number of fledglings per monitored pair, as well as the monitoring of marked individuals and the plumage colouration of parents and their offspring. One or more nesting-platforms may constitute a territory, which was considered the sampling unit used to record all data during the reproductive period. Therefore, this dataset^14^ provides ecological information about territorial occupancy, productivity, site fidelity and polymorphism, respectively (Fig. 1).

### Occupancy

(Table Occupancy^14^). Territory occupation was assessed each year from late March to early May. Occupancy was determined when signs of territorial or mating behaviour were observed, including courtship and territorial flights and responses (e.g. elicited vocalizations, approaches), copulation, nest material transfers, the presence of at least one freshly refurbished nest or direct evidence of reproduction. Since booted eagle individuals may use a different nest from that used previously in a given territory, we used each territory as sampling unit. The search for nests consisted of locating the territories of the breeding pairs during the courtship period, when species show strong intra- and interspecific territorial defence behaviour, and a subsequent search on foot to identify new nesting-platforms. When a new nest was found, its location was recorded by a GPS unit and incorporated in a geographical information system (GIS). All forested areas were inspected regardless of whether they were considered suitable nesting sites or not^40^.

The spatial scales of raptor monitoring include territories and nests. Territory is defined as “any stretch of forest containing one (usually) or several nests (up to seven) within less than 300 m from each other, which is defended by breeding pairs”, which is not to be confused with the foraging areas, the home range of booted eagles being up to 25 km from the nest^41,42^. A nest is defined as “a large platform constructed of twigs and leaves, and placed either between the trunk and the branches or on the branches of the trees“^27^. Although nest size may vary in different years, it tends to increase when birds repair nests with new material for nest reuse and diminish when nests are not used for long time (low nest occupancy); they may also deteriorate or be affected by adverse weather conditions^43^. Since booted eagle alternate territories and nests in successive years with other forest raptor species (common buzzard and northern goshawk), we also recorded the information about territorial and nest occupancy of these species. Therefore, we recorded information about nest building in the study period by the three forest raptor species (all three species construct nest of similar appearance and dimensions), when a species was observed using a brand-new nest. A nest was considered destroyed when the whole structure had fallen from the nest tree or the branch that sustained it, when the nest tree or nest branches were broken or had fallen, or when most of the nest material (80%) had deteriorated due to natural causes, mainly meteorological perturbations, resulting in a loss of structural integrity.

### Productivity

(Table Productivity^14^). When a territory was occupied, at least three visits were made to record productivity by climbing the nest tree or observing from a distance using binoculars (x10) or telescope (x20–60). Productivity was expressed as the number of fledglings per monitored pair, considering those which survived to about 45 days old^44^.

We estimated the egg laying date by backdating from the hatching date of the oldest chick, assuming an incubation period of 38 days^45^. The hatching date was estimated from the age of chicks according to plumage development, using as reference personal observations made in other nests and descriptions provided by Cramp and Simmons^46^, and backdating accordingly. Since, as mentioned above, booted eagle alternate territories and nests over the years with other forest raptor species (common buzzard and northern goshawk), as occupancy dataset we also recorded information of fledglings of these species. Whereas booted eagle females lay one or two eggs, buzzard females lay one to three eggs and goshawk females lay one to four eggs.

### Marked individuals and polymorphism

**Fig. 2:**
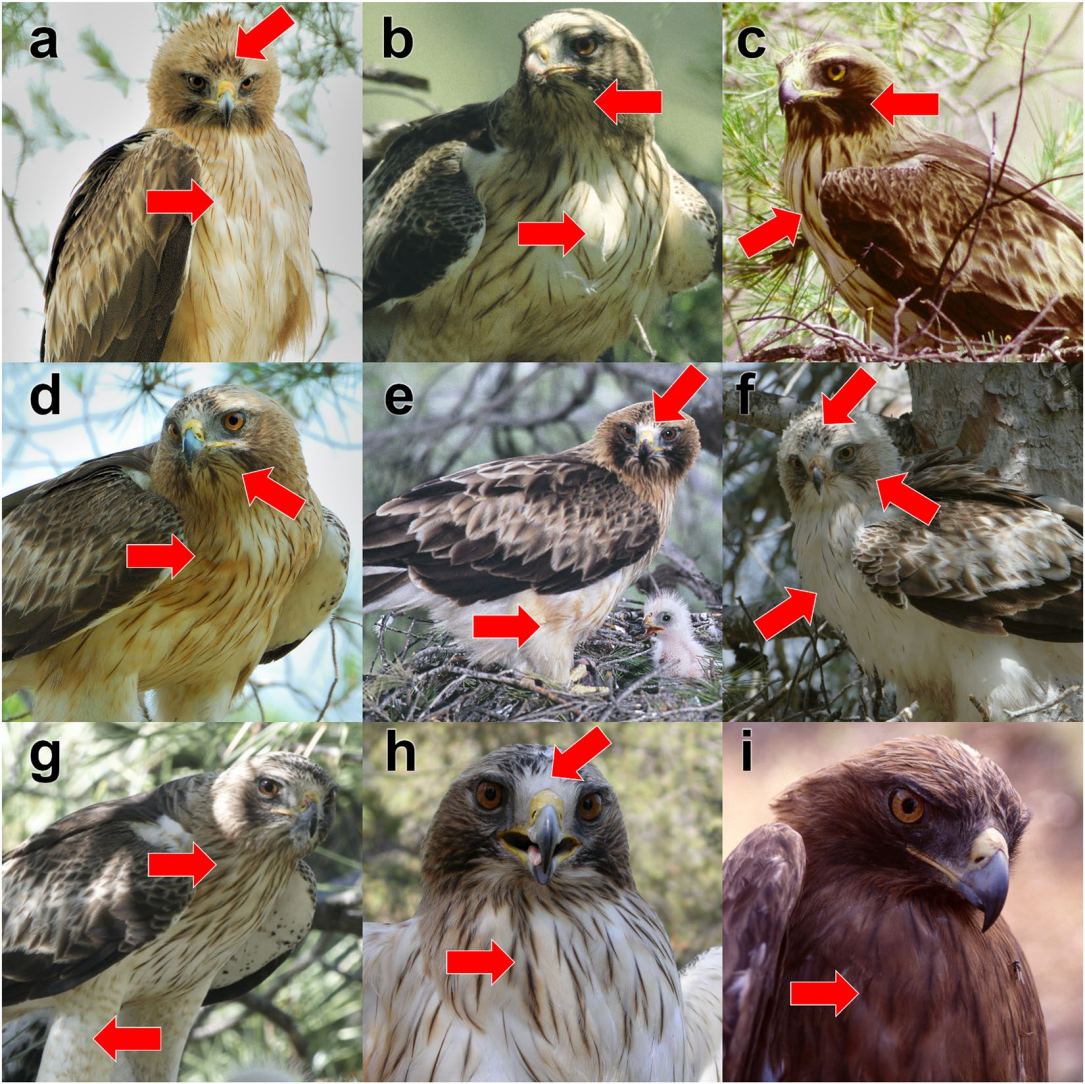
Examples of individually identified male (**a,f,h**) and female (**b–e,g,i**) booted eagles. Relevant plumage characteristics (red arrows) include cheeks, throats, breasts, foreheads and trousers. Individuals h and i were trapped for a radio-tracking study^41^. Photo credits: Carlos González Revelles (**a–g**) and José Francisco Calvo (**h,i**).

(Table Marked-individual and Table Polymorphism^14^, respectively). Regarding “Marked-individual” dataset, 86 breeding booted eagle individuals (48 females and 38 males) were identified by visual identification with certainty in 31 territories. Individuals were identified through direct observations, using schematic drawings or photographs (Fig. 2). The high variation in plumage colour, and especially tarsus pigmentation and head pattern^47^, allowed some booted eagles to be recognised from year to year. The sex of each individual was easily recognizable from its size and behaviour. Moreover, the plumage colouration for both members of a pair was recorded by direct observation. Regarding the “Polymorphism” dataset, we recorded fledgling polymorphism, considering the territory. Morph scoring followed the recognition scheme of Cramp and Simmons^46^, del Hoyo *et al*.^45^ and Forsman^48^, where two morphs are recognized (dark and pale); melanic individuals had a greater amount of eumelanic feathers.

These methods are expanded versions of descriptions in our related studies of territorial occupancy^34,36,43,44^ (see Usage Notes section).

## Data Records

**Fig. 3:**
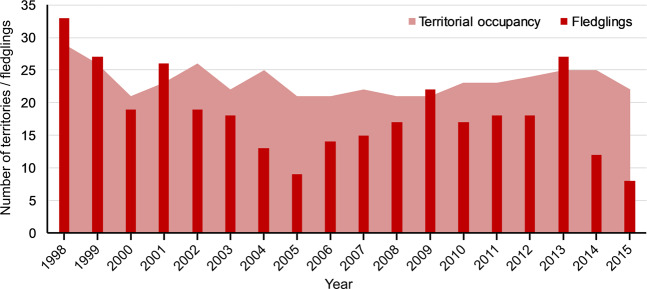
Summary of territorial occupancy and number of fledglings (productivity) during the study period for the booted eagle population in the Special Protection Area for birds (Sierras de Burete, Lavia y Cambrón “ES0000267”, south-eastern Spain).

The data are organized into 4 tables (.xlsx format; see Table 1 as a summary of data set) and have been deposited in the figshare repository^14^. Table Occupancy^14^ documents the contents and format for the complete dataset of territorial and nest occupancy, including the number of nests occupied by booted eagle, as well as the other two species that interchanged territories and reused nests in different reproductive years during the study period, the common buzzard and northern goshawk^43^. The average occupancy of booted eagle was 23.33 ± 2.28 breeding pairs per year (Fig. 3), while common buzzard and northern goshawk had a lower abundance, with an average occupancy of 6.78 ± 2.41 and 2.61 ± 1.14 breeding pairs per year, respectively. In total, 557 observations of occupancy were made for the three species, corresponding to 163 nests and 72 territories.

**Table 1:** Summary of the complete dataset generated and described in this study based on the long-term population monitoring of a raptor species, the booted eagle.

Data	Name of the table	Available format	Sample	Territory localization	Protocol	Timeframe period	Number of records	Structure of table
Table 1	Occupancy	Table in .xlsx	Nest	Number of territory	Census	1998–2015	1567	C1: number of the nest; C2: number of territory; C3-22: occupancy by years
Table 2	Productivity	Table in .xlsx	Nest	Number of territory	Census	1998–2015	600	C1: number of the nest; C2: number of territory; C3-22: fledglings by years
Table 3	Marked-individuals	Table in .xlsx	Individual	Number of territory	Census	1998–2009	187	C1: individual code; C2: sex; C3: number of territory; C4: year, C5: phenology; C6: fledglings, C7: individual experience, etc.
Table 4	Polymorphism	Table in .xlsx	Colour polymorphism	Number of territory	Census	1998–2013	373	C1: year; C2: number of territory; C3: fledglings; C4: male morph; C5: female morph; C6: phenology; C7:egg-laying; C8: fledgling pale morph; C9: fledgling dark morph

The table indicates the name of each dataset^14^, the type of sample, the time span during which the information was obtained, the total number of records available and the structure of the tables (column names) in the file in .xlsx format. Additional details available for recorded information are provided in the legend of each table in the .xlsx file.

**Fig. 4:**
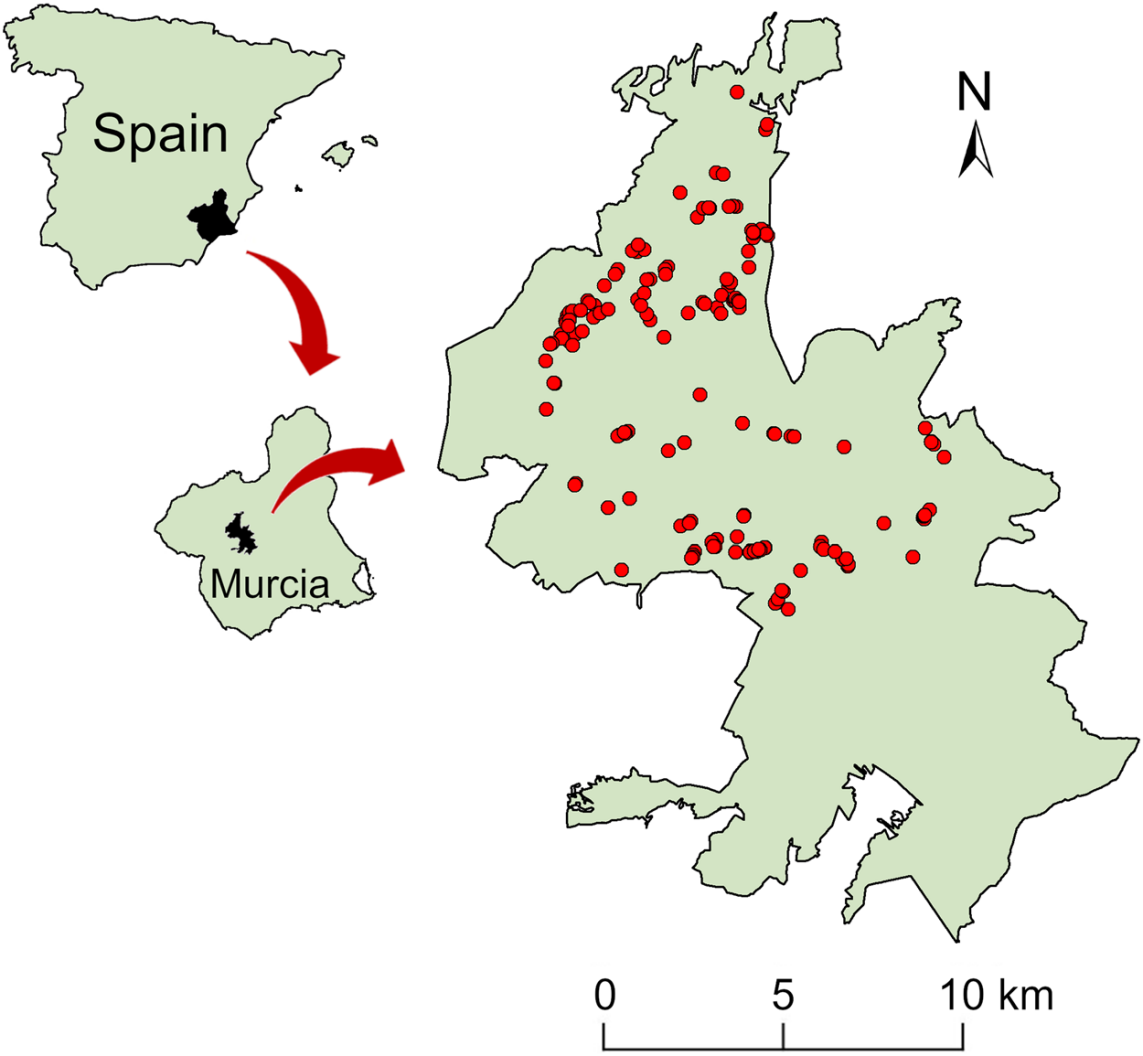
Distribution of all nesting platforms (*n* = 163) monitored in the Special Protection Area for birds (Sierras de Burete, Lavia y Cambrón “ES0000267”, in the Region of Murcia, south-eastern Spain) for the whole the study period (1998-2015).

Table Productivity^14^ compiles the number of fledglings of each occupied nest for the booted eagle during the study period, as well as that of the other two forest raptors alternating nests. In total, 435 fledglings (332 for booted eagle, 101 for common buzzard and 85 for northern goshawk) were recorded with certainty during the study period with an average productivity of 18.44 ± 6.57 fledglings per year for booted eagle (Fig. 3). Common buzzard and northern goshawk had lower productivity: 5.61 ± 3.87 and 4.72 ± 2.65 fledglings per year, respectively. Although the coordinates of the nests were not included in this dataset in order to protect the nests of forest raptor species, Fig. 4 shows the nest distribution in the study area for the whole the study period (1998–2015).

Table Marked-individuals^14^ includes the individuals of booted eagle identified by direct observations (48 females and 38 males) with a total of 187 observations, including information about their productivity, previous experience of individuals in nests, etc. (see details in Table 1) from 1998 to 2009.

**Fig. 5:**
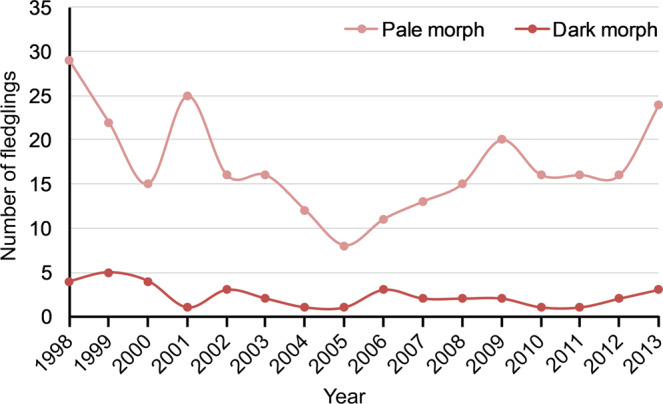
Summary of colour polymorphism in the number of fledglings of booted eagle during the study period (1998–2013) in the Special Protection Area for birds (Sierras de Burete, Lavia y Cambrón “ES0000267”, south-eastern Spain).

Finally, Table Polymorphism^14^ contains information about the different colour morph in parents and fledglings of booted eagle according to territory and year, including other variables (see details in Table 1), with a total of 373 observations from 1998 to 2013. The average for fledglings during the study period was 17.13 ± 5.55 pale morph fledglings and 2.32 ± 1.25 dark morph fledglings, proportions that were maintained across years (Fig. 5). The information provided in the dataset^14^ was analysed to study the different ecological processes involved in population ecology of booted eagle, which are described in Table 2 (see Usage Notes below).

**Table 2:** Summary of ecological topics studied based on the census of a booted eagle population, classifying the spatial scale, type of recorded data and main studies.

Ecological topic studied	Scale		Type of recorded data				Study
	Territory	Nest	Occupancy	Productivity	Marked-Individuals	Polymorphism	
Environmental characteristics conditioning territorial occupancy	x		x	x			Martínez *et al*.^34^
Interannual variations of reproductive parameters	x			x			Martínez *et al*.^44^
Territorial occupancy in relation to occupancy parameters	x		x				Pagán *et al*.^57^
Interspecific territorial occupancy	x		x				Jiménez-Franco *et al*.^36^
Factor conditioning site fidelity of booted eagle	x		x	x	x		Jiménez-Franco *et al*.^61^
Temporal effects on territory and nest patterns	x		x	x			Martínez *et al*.^43^
Territorial and nest occupancy patterns	x	x	x	x			Jiménez-Franco *et al*.^58^
Nest lifespan		x	x	x			Jiménez-Franco *et al*.^59^
Reproductive output of booted eagle and influence of precipitation	x		x	x			Bosch *et al*.^60^
Polymorphic characteristics of booted eagle and productivity	x			x		x	Martínez *et al*.^62^
Nest conservation in forestry practices and occupancy		x	x	x			Jiménez-Franco *et al*.^26^
Heritability of polymorphism in booted eagle	x					x	Bosch *et al*.^63,64^

## Technical Validation

The long-term monitoring program, whose data the three forest raptor species are described in this study, was designed by researchers José F. Calvo and José E. Martínez. This research line has led to the development of three Doctoral Theses^49–51^ and one Bachelor Thesis^52^. We assumed that the locations of all nests and territories had been known since 1998, when the detection of occupation was perfect (*p* = 1), since an intensive search was carried out to locate them in 1996 and 1997, and subsequent searches were performed to find new nests each breeding period. Name of territory locations were validated and checked with local descriptions in regional maps of scale 1:25,000. In order to validate the productivity each nest was visited at least three times during the reproductive period. Productivity validation followed the criteria described by Steenhof^53^ and followed the usual methods used for forest raptor census^54^. As regards the identification of individuals, this is a difficult task and usually requires capture and marking. However, capture is costly and considered harmful in some raptors^55^, so we followed the method outlined by Krüger for individual identification^56^. Moreover, only easily identifiable individuals (and usually the partner) were selected in different territories.

## Usage Notes

The data provided are useful for analysing different ecological processes (see Table 2). For example, based on the occupancy information, it is possible to analyse the factors conditioning territorial occupancy^34,57^, detect processes such as colonization, abandonment, persistence and species alternation in territories^36^, processes of territory creation, new establishments, nest creation or destruction and processes such as the use of nests as location cues^58^. Furthermore, the alternation of species in the same nests can be analysed to study interspecific relationships^59^. This knowledge may be applied to the conservation of raptor populations and forest management^26,43^.

In the case of productivity information, the data help in the long-term study of the breeding biology^44^, as well as the relation of breeding success with rainfall^60^. Moreover, it is useful to relate reproductive output with nest building and nest reuse^58^ and the influence of nest reuse on breeding output^59^. Moreover, the identification of booted eagle’s marked individuals provides information about the site fidelity in relation to the previous breeding season^61^. Finally, with the plumage coloration data provided for booted eagle, the possible connection between parents colour polymorphism and brood size can be studied^62^, and the inheritance patterns of the particular colour morph^63,64^.

## References

[1] Lindenmayer DB (2012). Value of long‐term ecological studies. Austral Ecol..

[2] Sutter RD, Wainscott SB, Boetsch JR, Palmer CJ, Rugg DJ (2015). Practical guidance for integrating data management into long-term ecological monitoring projects. Wildl. Soc. Bull..

[3] Nichols JD, Williams BK (2006). Monitoring for conservation. Trends Ecol. Evol..

[4] Applegate RD (2015). The importance of data management in wildlife conservation. Wildl. Soc. Bull..

[5] Perrig PL, Lambertucci SA, Donadio E, Padró J, Pauli JN (2019). Monitoring vultures in the 21 st century: The need for standardized protocols. J. Appl. Ecol..

[6] Dornelas M (2018). BioTIME: A database of biodiversity time series for the Anthropocene. Glob. Ecol. Biogeogr..

[7] Johnson, D. H., Nieuwenhuyse, D. V. & Nelson, M. D. European forest owls: Population status, trends, conservation and monitoring. In *Ecology and conservation of European forest-dwelling raptors*. (eds. Zuberogoitia, I. & Martínez, J. E.) 40–49. Diputación Foral de Vizcaya, Bilbao (2011).

[8] Sergio F (2018). Raptor monitoring: challenges and benefits. Bird Study.

[9] Zuberogoitia I (2018). Population trends of Peregrine Falcon in Northern Spain – Results of a long-term monitoring project. Ornis Hungarica.

[10] Martínez-Martí C, Jiménez-Franco MV, Royle JA, Palazón JA, Calvo JF (2016). Integrating occurrence and detectability patterns based on interview data: a case study for threatened mammals in Equatorial Guinea. Sci. Rep..

[11] Dornelas M (2019). A balance of winners and losers in the Anthropocene. Ecol. Lett..

[12] Tapia, L. & Zuberogoitia, I. Breeding and Nesting Biology in Raptors. In *Birds of Prey* 63–94. Springer, Cham., (2018).

[13] Nordell, C. J. & Franke, A. Systematic data management. In *Applied raptor ecology: essentials from Gyrfalcon research*. (eds. Anderson, D. L., McClure, C. J. W. & Franke, A.) 75–90. The Peregrine Fund, Boise, Idaho, USA (2017).

[14] Jiménez-Franco MV, Martínez JE, Pagán I, Calvo JF (2020). figshare.

[15] Helander B, Bignert A, Asplund L (2008). Using raptors as environmental sentinels: monitoring the white-tailed sea eagle *Haliaeetus albicilla* in Sweden. Ambio.

[16] García-Fernández AJ, Calvo JF, Martínez-López E, María-Mojica P, Martínez JE (2008). Raptor ecotoxicology in Spain: a review on persistent environmental contaminants. Ambio.

[17] Martínez-López E (2005). Cadmium in feathers of adults and blood of nestlings of three raptor species from a nonpolluted Mediterranean forest, southeastern Spain. Bull. Environ. Contam. Toxicol..

[18] Martínez-López E (2007). Organochlorine residues in booted eagle (*Hieraaetus pennatus*) and goshawk (*Accipiter gentilis*) eggs from southeastern Spain. Environ. Toxicol. Chem..

[19] Martínez-López E (2009). Changes in blood pesticide levels in booted eagle (*Hieraaetus pennatus*) associated with agricultural land practices. Ecotoxicol. Environ. Saf..

[20] Sergio F, Newton I, Marchesi L (2005). Top predators and biodiversity. Nature.

[21] Witmer GW (2005). Wildlife population monitoring: some practical considerations. Wildl. Res..

[22] León-Ortega M, Jiménez-Franco MV, Martínez JE, Calvo JF (2017). Factors influencing territorial occupancy and reproductive success in a Eurasian Eagle-owl (*Bubo bubo*) population. PloS One.

[23] Seamans ME, Gutiérrez RJ (2007). Habitat selection in a changing environment: the relationship between habitat alteration and spotted owl territory occupancy and breeding dispersal. The Condor.

[24] Martínez, J. E., Pagán, I., Jiménez-Franco, M. V. & Calvo, J. F. Ecology of the booted eagle in semiarid Mediterranean landscapes. In *Ecology and conservation of European forest-dwelling raptors* (eds. Zuberogoitia, I. & Martínez, J. E.) 226–233. Diputación Foral de Bizkaia. Bilbao (2011).

[25] Santangeli A, Högmander J, Laaksonen T (2013). Returning white‐tailed eagles breed as successfully in landscapes under intensive forestry regimes as in protected areas. Anim. Conserv..

[26] Jiménez-Franco MV (2018). Nest sites as a key resource for population persistence: A case study modelling nest occupancy under forestry practices. PLoS One.

[27] Petty, S. J. *Ecology and Conservation of Raptors in Forests*. London: Forestry Commission Bulletin, 118 (1998).

[28] Björklund, H., Valkama, J., Saurola, P. & Laaksonen, T. Evaluation of artificial nests as a conservation tool for three forest‐dwelling raptors. *Anim. Conserv.* **16**, 546–555 (2013).

[29] García-Dios, I. S. Aguililla calzada – *Hieraaetus pennatus*. In *Enciclopedia Virtual de los Vertebrados Españoles*. (Eds. Salvador, A. & Morales, M. B.). Museo Nacional de Ciencias Naturales, Madrid. http://www.vertebradosibericos.org/ (2014).

[30] Martínez, J. E. & Calvo, J. F. Prey partitioning between mates in breeding Booted Eagles (*Hieraaetus pennatus*). *J. Raptor Res.* **39**, 159–163 (2005).

[31] Martínez, J.E., Cremades, M., Pagán, I. & Calvo, J.F. Diet of Booted Eagles (*Hieraaetus pennatus*) in Southeastern Spain. In *Raptors Worldwide* (eds. Chancellor, R. D. & Meyburg, B. U.) 593-599. WWGBP/MME. Budapest, Hungary (2004).

[32] Robledano, F., Calvo, J. F. & Aledo, E. *Libro Rojo de los Vertebrados de la Región de Murcia*. Consejería de Industria y Medio Ambiente. Región de Murcia (2006).

[33] Martínez JE, Zuberogoitia I, Jiménez-Franco MV, Mañosa S, Calvo JF (2016). Spatio-temporal variations in mortality causes of two migratory forest raptors in Spain. Eur. J. Wildl. Res..

[34] Martínez JE, Pagán I, Calvo JF (2006). Factors influencing territorial occupancy and reproductive output in the Booted Eagle *Hieraaetus pennatus*. Ibis.

[35] Krüger O (2002). Interactions between common buzzard *Buteo buteo* and goshawk *Accipiter gentilis*: trade-off revealed by a field experiment. Oikos.

[36] Jiménez-Franco MV, Martínez JE, Calvo JF (2011). Territorial occupancy dynamics in a forest raptor community. Oecologia.

[37] Tapia, L. In *Enciclopedia Virtual de los Vertebrados Españoles*. (eds. Salvador, A. & Bautista, L. M.). Museo Nacional de Ciencias Naturales, Madrid, http://www.vertebradosibericos.org/ (2010).

[38] Dunning, J. B. J. *CRC Handbook of Avian Body Masses*. *Second Edition*. *CRC Press*. *NY*. (2007).

[39] Sánchez-Zapata JA, Calvo JF (1999). Raptor distribution in relation to landscape composition in semi-arid Mediterranean habitats. J. Appl. Ecol..

[40] Hubert, C. Nest-site habitat selected by Common buzzard (*Buteo buteo*) in southwestern France. *J. Raptor Res.* **27**, 102–105 (1993).

[41] Martínez JE, Pagán I, Palazón JA, Calvo JF (2007). Habitat Use of Booted Eagles (*Hieraaetus pennatus*) in a Special Protection Area: Implications for Conservation. Biodivers. Conserv..

[42] López-López, P., Martínez, J. E. & Calvo, J. F. Ecología espacial en el periodo reproductor. In *Migración y ecología espacial de la población española de águila calzada* (eds. Urios, V., Bermejo, A., Vidal-Mateo, J. & De la Puente, J.) 33–42. Monografía n. °2 del programa Migra. SEO/BirdLife. Madrid (2017).

[43] Martínez JE, Jiménez-Franco MV, Zuberogoitia I, León-Ortega M, Calvo JF (2013). Assessing the short-term effects of an extreme storm on Mediterranean forest raptors. Acta Oecologica.

[44] Martínez JE, Pagán I, Calvo JF (2006). Interannual variations of reproductive parameters in a booted eagle (*Hieraaetus pennatus*) population: the influence of density and laying date. J. Ornithol..

[45] del Hoyo, J., Elliot, A. & Sargatal, J. *Handbook of the birds of the world*, *vol. 2*. *New world vultures to guineafowl*. **2**, Lynx Edicions, Barcelona (1994).

[46] Cramp S, Simmons KEL (1980). Handbook of the birds of Europe, the Middle East and North Africa..

[47] Bretagnolle V, Thibault J-C, Dominici J-M (1994). Field identification of individual Ospreys using head marking pattern. J. Wildl. Manage..

[48] Forsman, D. *The raptors of Europe and The MiddleEast. A handbook of field identification*. T & AD Poyser, London, UK (1999).

[49] Martínez, J. E. Ecología del Águila Calzada (*Hieraaetus pennatus*) en ambientes mediterráneos. Tesis Doctoral. Universidad de Murcia. Murcia (2002).

[50] Pagán, I. *Patrones de ocupación territorial del Aguililla Calzada en sistemas forestales mediterráneos*. Tesis Doctoral. Universidad de Murcia. Murcia (2008).

[51] Jiménez-Franco, M. V. *Modelos de ocupación territorial en rapaces forestales*. Tesis Doctoral. Universidad de Murcia. Murcia (2014).

[52] Jiménez-Franco, M. V. *La experiencia reproductiva previa como factor codicionante de la ocupación y fidelidad territorial del aguililla calzada (Hieraaetus pennatus)*. Tesina de Licenciatura. Universidad de Murcia. Murcia (2010).

[53] Steenhof, K. Assessing raptor reproductive success and productivity. In *Raptor management techniques manual* (eds. Giron Pendleton, B. A., Millsap, B. A., Cline, K. W. & Bird, D. M.) 157–170. Washington: National Wildlife Federation (1987).

[54] Fuller, M. R. & Mosher, J. A. Raptor survey techniques. In *Raptor management techniques manual* (eds. Giron Pendleton, B. A., Millsap, B. A., Cline, K. W. & Bird, D. M.) 157–170. Washington: National Wildlife Federation (1987).

[55] Bird, D. M. & Bildstein, K. L. *Raptor research and management techniques. Handcock House* (2007).

[56] Krüger O (2002). Dissecting common buzzard lifespan and lifetime reproductive success: the relative importance of food, competition, weather, habitat and individual attributes. Oecologia.

[57] Pagán I, Martínez JE, Calvo JF (2009). Territorial occupancy and breeding performance in a migratory raptor do not follow ideal despotic distribution patterns. J. Zool..

[58] Jiménez-Franco MV, Martínez JE, Calvo JF (2014). Patterns of nest reuse in forest raptors and their effects on reproductive output. J. Zool..

[59] Jiménez-Franco, M. V., Martínez, J. E. & Calvo, J. F. Lifespan analyses of forest raptor nests: Patterns of creation, persistence and reuse. *PLoS One* **9,** e93628 (2014). 10.1371/journal.pone.0093628 PMC3981714 24717935

[60] Bosch J, Martínez JE, Calvo JF, Zuberogoitia I, Jiménez-Franco MV (2015). Does rainfall affect the productivity of the Booted Eagle (*Aquila pennata*) during the breeding period in Mediterranean environments?. J. Ornithol..

[61] Jiménez-Franco MV, Martínez JE, Pagán I, Calvo JF (2013). Factors determining territory fidelity in a migratory forest raptor, the Booted Eagle *Hieraaetus pennatus*. J. Ornithol..

[62] Martínez JE (2016). Colour morph does not predict brood size in the Booted Eagle. Ornis Fenn..

[63] Bosch J (2019). Colour plumage polymorphism in the Booted Eagle: inheritance pattern and temporal stability of the morph frequencies. J. Zool..

[64] Bosch, J. *et al*. Evidence of non-random mating in a colour polymorphic raptor, the Booted Eagle. *J. Ornithol*. 10.1007/s10336-020-01763-y (2020).

